# Inhibition of the NEMO/IKKβ association complex formation, a novel mechanism associated with the NF-κB activation suppression by *Withania somnifera’s* key metabolite withaferin A

**DOI:** 10.1186/1471-2164-11-S4-S25

**Published:** 2010-12-02

**Authors:** Abhinav Grover, Ashutosh Shandilya, Ankita Punetha, Virendra S Bisaria, Durai Sundar

**Affiliations:** 1 Department of Biochemical Engineering and Biotechnology, Indian Institute of Technology (IIT) Delhi, Hauz Khas, New Delhi 110016, India; 2 Supercomputing Facility for Bioinformatics and Computational Biology, Indian Institute of Technology (IIT) Delhi, Hauz Khas, New Delhi 110016, India

## Abstract

**Background:**

Nuclear Factor kappa B (NF-κB) is a transcription factor involved in the regulation of cell signaling responses and is a key regulator of cellular processes involved in the immune response, differentiation, cell proliferation, and apoptosis. The constitutive activation of NF-κB contributes to multiple cellular outcomes and pathophysiological conditions such as rheumatoid arthritis, asthma, inflammatory bowel disease, AIDS and cancer. Thus there lies a huge therapeutic potential beneath inhibition of NF-κB signalling pathway for reducing these chronic ailments. *Withania somnifera*, a reputed herb in ayurvedic medicine, comprises a large number of steroidal lactones known as withanolides which show plethora of pharmacological activities like anti- inflammatory, antitumor, antibacterial, antioxidant, anticonvulsive, and immunosuppressive. Though a few studies have been reported depicting the effect of WA (withaferin A) on suppression of NF-κB activation, the mechanism behind this is still eluding the researchers. The study conducted here is an attempt to explore NF-κB signalling pathway modulating capability of *Withania somnifera’s* major constituent WA and to elucidate its possible mode of action using molecular docking and molecular dynamics simulations studies.

**Results:**

Formation of active IKK (IκB kinase) complex comprising NEMO (NF-κB Essential Modulator) and IKKβ subunits is one of the essential steps for NF-κB signalling pathway, non-assembly of which can lead to prevention of the above mentioned vulnerable disorders. As observed from our semi-flexible docking analysis, WA forms strong intermolecular interactions with the NEMO chains thus building steric as well as thermodynamic barriers to the incoming IKKβ subunits, which in turn pave way to naive complex formation capability of NEMO with IKKβ. Docking of WA into active NEMO/IKKβ complex using flexible docking in which key residues of the complex were kept flexible also suggest the disruption of the active complex. Thus the molecular docking analysis of WA into NEMO and active NEMO/IKKβ complex conducted in this study provides significant evidence in support of the proposed mechanism of NF-κB activation suppression by inhibition or disruption of active NEMO/IKKβ complex formation being accounted by non-assembly of the catalytically active NEMO/IKKβ complex. Results from the molecular dynamics simulations in water show that the trajectories of the native protein and the protein complexed with WA are stable over a considerably long time period of 2.6 ns.

**Conclusions:**

NF-κB is one of the most attractive topics in current biological, biochemical, and pharmacological research, and in the recent years the number of studies focusing on its inhibition/regulation has increased manifolds. Small ligands (both natural and synthetic) are gaining particular attention in this context. Our computational analysis provided a rationalization of the ability of naturally occurring withaferin A to alter the NF-κB signalling pathway along with its proposed mode of inhibition of the pathway. The absence of active IKK multisubunit complex would prevent degradation of IκB proteins, as the IκB proteins would not get phosphorylated by IKK. This would ultimately lead to non-release of NF-κB and its further translocation to the nucleus thus arresting its nefarious acts. Conclusively our results strongly suggest that withaferin A is a potent anticancer agent as ascertained by its potent NF-κB modulating capability. Moreover the present MD simulations made clear the dynamic structural stability of NEMO/IKKβ in complex with the drug WA, together with the inhibitory mechanism.

## Background

NF-κB (Nuclear Factor kappa B) is a ubiquitous transcription factor involved in the regulation of cell signaling responses. It is a key regulator of cellular processes involved in the immune response, differentiation, cell proliferation, and apoptosis [1,2]. NF-κB is secreted predominantly in cytoplasm in the form of an inactive complex with IκB inhibitor proteins. Binding to IκB (Inhibitor of kappa B) prevents NF-κB:IκB complex from translocating to the nucleus, thereby maintaining NF-κB in an inactive state. NF-κB signalling is generally considered to occur through NF-κB activation being inititated by stimuli like proinflammatory cytokine TNF (tumor necrosis factor) alpha and bacterial lipopolysaccharide (LPS). Signalling pathways lead to activation of the beta subunit of the IKK (IκB kinase) complex, which then phosphorylates IκB proteins leading to their degradation and subsequent release of NF-κB. The freed NF-κB dimers translocate to the nucleus where it binds to the target genes. The constitutive activation of NF-κB contributes to multiple cellular outcomes and pathophysiological conditions such as rheumatoid arthritis, asthma, inflammatory bowel disease [3], AIDS [4] and cancer [5]. Thus there lies a huge therapeutic potential beneath inhibition of NF-κB signalling pathway for reducing menace of these chronic ailments [6].

Degradation of IκB is a tightly regulated event that is initiated upon specific phosphorylation by activated IKK. IKK is a multisubunit complex that contains two kinase subunits, IKKα (IKK1) and IKKβ (IKK2), and a regulatory subunit, NEMO (NF-κB Essential Modulator) or IKKc [7]. In the classical NF-κB signalling pathway, IKKβ is both necessary and sufficient for phosphorylation of IκBα on Ser 32 and Ser 36, and IκBβ on Ser 19 and Ser 23. Thus inhibition of NEMO/IKKβ complex assembly by employment of small molecule inhibitors can offer a modest mode of inhibition of NF-κB activation while providing additional favors of oral administration and decreased immunogenicity.

*Withania somnifera*, also known as “ashwagandha”, “Queen of Ayurveda”, “Indian ginseng”, and “winter cherry”, has been an important herb in the Ayurvedic and indigenous medical systems for more than 3,000 years [8]. Its roots have been used as herb remedy to treat a variety of ailments and to promote general wellness. It has received much attention in recent years due to the presence of a large number of alkaloids and steroidal lactones known as withanolides [9,10]. Many of Ashwagandha’s pharmacological activities have been attributed to two primary withanolides: withaferin A (WA) and withanolide D. The principal withanolide in the Indian variety of the plant is WA. This drug is known to have anti-inflammatory [11], antitumor [12], antibacterial [13], antioxidant [14], anticonvulsive [15], and immunosuppressive properties [16]. It has the potential to increase tumor sensitization to radiation and chemotherapy while reducing some of the most common side effects of these conventional therapies [17]. Long-term effects of *W. somnifera* on adjuvant-induced arthritis in rats have also been reported [18]. Most recently, these were shown to potentiate apoptosis of tumor cells by suppression of NF-κB activation [19-21], protect against UV-induced skin cancer [22] and enhance neurite regeneration and memory [23,24]. Thus, many studies have been reported depicting the effect of WA on suppression of NF-κB activation, but the mechanism behind this effect is still eluding the researchers. The study conducted here is an attempt to elucidate a possible mode of action of *Withania somnifera’s* major constituent WA on NF-κB signalling pathway using molecular docking studies.

### Structural aspects of NEMO/IKKβ association domain

**Figure 1:**
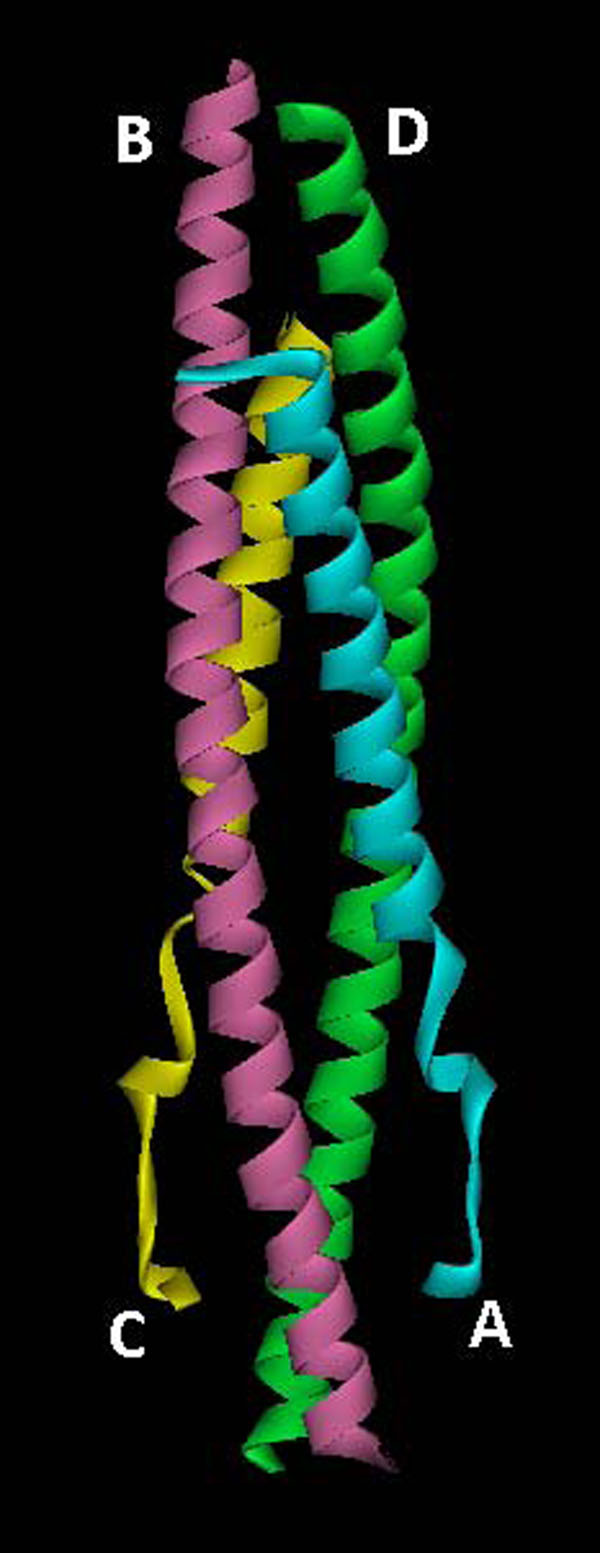
**Ribbon structure of NEMO/IKKβ complex.** Chains B (pink) and D (green) constitutes the NEMO domain while chains A (cyan) and C (yellow) represent secondary structure of IKKβ.

The structural features of the receptor macromolecule [PDB: 3BRV] have been described in detail elsewhere [25] by the depositors of the crystal structure to the Protein Data Bank. Briefly, the protein is a 4-helix bundle of NEMO and IKKβ domains each consisting of two chains B, D and A, C respectively as shown in Figure 1. NEMO density extends from residues 49 to 109 in chain B and from 49 to 109 in chain D. The IKK peptide density extends from residues 705 to 743 in chain A and from residues 701-744 in chain C. N and C termini of NEMO chains B and D form dimerization patches on the ends where the two IKK peptides attach themselves. The IKK peptides are almost helical except for an unwound stretch ranging from 732 to 742. The interactions between side chain tryptophans and main chain amides cause constriction in the backbone resulting in this unwounding region. Each of the IKKβ peptide chain associate loosely with the corresponding NEMO chains at their N-terminus but they are stringently attached to the two NEMO chains at the C-terminus. The authors have assigned three regions within the IKKβ peptide, designated as helical (705–731), linker (732–736), and the NBD (NEMO Binding Domain) (737–742). Residues 85–101 of dimeric NEMO form a flat slit paving way to two broad and extensive IKK-binding pockets; each pocket being occupied by the IKK peptide linker and the NBD. The IKK peptide forms intermolecular hydrogen-bond interactions (Ser85:Q730 and Glu89:S733) with NEMO in the NEMO’s specificity pocket. Three large IKK side chains inside the NEMO pocket which form consolidated intermolecular hydrophobic interactions (Leu93:F734, Phe92:T735, Met94:F734, Phe97:W739, Ala100:W741, and Arg101:W741) are responsible for formation of NEMO-IKKβ complex.

## Methods

### Ligand and receptors

**Figure 2:**
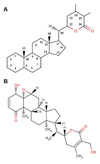
**Structures of withanolides.** (A) Withaferin A falls under the family of compounds known as withanolides which are a group of naturally occurring C28- steroidal lactones built on an intact or rearranged ergostane framework, in which C-22 and C- 26 are appropriately oxidized to form a six-membered lactone ring. The basic structure is designated as the withanolide skeleton defined as a 22-hydroxyergostan-26-oic acid-26,22-lactone. (B) Structure of withaferin A

The crystal structure of the NEMO/IKKβ association domain [PDB: 3BRV] was obtained from the Protein Data Bank (PDB) [26]. The crystal structure contained many missing atoms which were supplemented by the repairCommands module of AutoDock. Before docking, the protein crystal structure was cleaned by removing the water molecules. H-atoms were added to these target proteins for correct ionization and tautomeric states of amino acid residues. The modified structure so obtained was used for all the flexible docking studies while only NEMO chains were used for performing semi-flexible dockings. The ligand molecule withaferin A [PubChem:265237] was retrieved from NCBI-PubChem Compound database [27]. Figure 2 shows the basic skeleton of withanolides along with the structure withaferin A. The energy of the ligand molecule and receptors were minimized in Steepest Descent and Conjugate Gradient methods using Accelrys Discovery Studio (Version 1.7, Accelrys Software Inc.), the most comprehensive suite of modeling and simulation solutions for drug discovery available. Each of the minimization methods were carried out with CHARMm force field.

### Semi-flexible docking

AutoDock 4.0 suite was used as molecular-docking tool in order to carry out the docking simulations [28]. Several studies report the comparison of AutoDock with various docking programs. AutoDock has been found to be able to locate docking modes that are consistent with X-ray crystal structures [29,30]. AutoDock helps to simulate interactions between substrates or drug candidates as ligands and their macromolecular receptors of known three dimensional structures, allowing ligand flexibility described to a full extent elsewhere [28]. In our docking simulations we first used only the NEMO domain of the NEMO-IKKβ association domain structure for performing semi-flexible docking, with the ligand WA made flexible while keeping the receptor macromolecule being rigid. Flexibility of the ligand helps it explore six spatial degrees of freedom for rotation and translation and an arbitrary number of torsional degrees of freedom. A random perturbation to each is applied at each time step, and the interaction energy was evaluated for the new location and conformation [31].

### Flexible docking

One of the novel features of AutoDock 4.0 allows side chains in the protein as well as in the ligand to be flexible. Thus flexibility of receptor molecule was also exploited in docking studies by making use of AutoDock flexres scripts. It is worth assumable that the incoming ligand arriving at the binding sites of the two chains would try to make its own associations with the residues in order to minimize the energy of the system. To facilitate this notion, the key residues of NEMO and IKKβ chains which form H-bonds with corresponding residues in the two chains as reported [25] were made flexible in order to observe their mode of interactions with the ligand and to account for the subsequent rearrangements. The peptide bonds of the amino acids chosen to be flexible were kept “inactive” or non-rotatable. The Graphical User Interface program "AutoDock Tools" was used to prepare, run, and analyze the docking simulations. Protein was prepared for docking simulations by assigning of Kollman united atom charges, solvation parameters and polar hydrogens to the receptor PDB file. Water molecules were removed from its PDB file to make it a free receptor. Since ligands are not peptides, Gasteiger charge was assigned and then nonpolar hydrogens were merged. AutoDock assigns the rigid roots to the ligand automatically saving time as compared to manual picking. Five bonds in the ligand were made “active” or rotatable. Atomic solvation parameters were assigned to the receptor using default parameters.

### Grid design

Auto-Tors, an auxiliary program using Interactive queries to define rotatable torsion angles, is used to assign all rotatable dihedrals in the ligands and to remove non-polar hydrogen atoms, uniting their partial charges with their bonded carbon atoms. AutoGrid, which calculates grids of interaction energy based on the interaction of the ligand atom probes with receptor target, is used to obtain the grid maps required prior to docking. Each probe consists of an atom type present in the ligand being docked. The pre-calculated grid maps store the potential energy arising from the interaction with the macromolecule. The user defined three dimensional grid must surround the region of interest in the macromolecule, and the ligand was limited to this search space during docking. In the present study, the location and dimensions of the grid box are chosen such that it incorporates the amino acid stretch (85-101) of NEMO domain involved in binding with the IKKβ chains for the formation of active IKK complex. The energy scoring grid was prepared as a 60, 60, and 60 A° (x, y, and z) cube. The spacing between grid points was 0.375 angstroms.

### The genetic algorithm

The Lamarckian Genetic Algorithm (LGA) was chosen to search for the best conformers. Using mathematical concepts designed to simulate the conditions influencing biological evolution, genetic algorithms are able to search conformational space by "mutating" a ligand in order to find its lowest energy conformation in the "environment" of a fixed protein. Searches driven by this energy funnelling have been shown to provide a good indication as to the optimum protein-ligand interactions and therefore conveying the structure most likely to be found *in vivo*[32]. The default parameters for the Lamarckian genetic algorithm [28] were used as the search protocol except for the maximum number of energy evaluations, which were changed to 2.5 million. During the docking process, a maximum of 20 conformers was considered for each compound. The population size was set to 150 and the individuals were initialized randomly. Maximum number of generations were kept as 1000, maximum number of top individual that automatically survived set to 1, mutation rate of 0.02, crossover rate of 0.8. Step sizes for translations, quaternions and torsions were kept same as defaults. An efficient and accurate energy assessment of the ligand conformations is as important to the success of a docking simulation as the power of the search algorithm. AutoDock uses a variation on the AMBER'95 force field [33] with terms empirically determined by linear regression analysis from a set of protein-ligand complexes with known binding constants [28,34]. Gibbs Free energy (ΔG) is calculated as a sum of six energy terms of dispersion/repulsion, hydrogen bonding, electrostatic interactions, deviation from covalent geometry, internal ligand torsional constraints, and desolvation effects.

### Selection and representation of docking modes

AutoDock reports the best docking solution (lowest docked free energy) for each GA run and also performs a cluster analysis in which the total number of clusters and the rank of each docking mode (cluster rank) are reported. Docking modes were selected on the basis of two criteria: ligand’s proximity to the N-terminal Glutamine and extent of its interactions with the IKKα and IKKβ interacting hydrophobic amino acid side chains of NEMO. For a 10 GA run there would be up to 10 total docking modes from which the lowest energy-docking mode was chosen that met the above two criteria. All the AutoDock docking runs were performed in Intel Core 2 Duo P8400 CPU @ 2.26 GHz of Sony origin, with 3 GB DDR RAM. AutoDock 4.0 was compiled and run under Windows VISTA operating system. The output from AutoDock and all modeling studies as well as images were rendered with PyMOL [35] and Accelerys ViewerLite 5.0. PyMOL was used to calculate the distances of hydrogen bonds as measured between the hydrogen and its assumed binding partner.

### Confirmation of the docking results

The docking results obtained using AutoDock were also confirmed using ParDOCK [36] , which is an all atom energy based monte carlo docking protocol. Docking using ParDOCK requires a reference complex (target protein bound to a reference ligand) and a candidate molecule along with specific mention of the centre of mass of the cavity on which the ligand is to be docked.

### MD simulations in water

The AMBER v.10 package [37]was used to prepare the protein and the ligand files as well as for the Molecular Dynamics (MD) simulations. The binding complex of NEMO/IKKβ/WA obtained using ParDOCK and the free protein simulated in this study were neutralized by adding appropriate number of sodium counter-ions and was solvated in a octahedron box of TIP3P water with a 10 Å distance between the protein surface and the box boundary [38]. The partial atomic charges for the ligand were obtained using “antechamber” [39] module of Amber.

The energy minimization and MD simulations of NEMO/IKKβ and its complex with WA were carried out with the aid of the SANDER module of the AMBER 10 program. First of all, the simulated binding complex was effected with a 1000 step minimization using the steepest descent algorithm followed by a 2000 step minimization using conjugate gradient to remove bad steric contacts. Topology and parameter files for the protein were generated using “ff03” and for the drug using “gaff” based on the atom types of the force field model developed by Cornell et al [33]. Then the system was equilibrated beginning with the protein atom restrained simulations having 20 ps equilibration dynamics of the solvent molecules at 300 K and a harmonic potential with a 10 kcal/mol restraint force. Next step involved the equilibration of the solute molecules with a fixed configuration of the solvent molecules in which the system was slowly heated from T = 10 to 300 K in three intervals of 20 ps each. The entire system was then equilibrated at 300 K for 70 ps before a sufficiently long MD simulation (for 2.6 ns) at room temperature. The MD simulations were performed with a periodic boundary condition in the NPT ensemble at T=298.15 K with Berendsen temperature coupling [40] and constant pressure P=1 atm with isotropic molecule-based scaling . The SHAKE algorithm [41] was applied to fix all covalent bonds containing hydrogen atoms. We used a time step of 2 fs and a nonbond-interaction cutoff radius of 10 A°. The Particle Mesh Ewald (PME) method [42] was used to treat long-range electrostatic interactions. The coordinates of the trajectory was sampled every 1 ps for analysis of the energy stabilization and RMSD values of the protein as well as that of the complex. MD simulations were performed on a 320 processors SUN Microsystems clusters at Supercomputing Facility (SCFBio) at IIT Delhi.

## Results and discussion

### Semi-flexible docking of WA into NEMO

**Figure 3:**
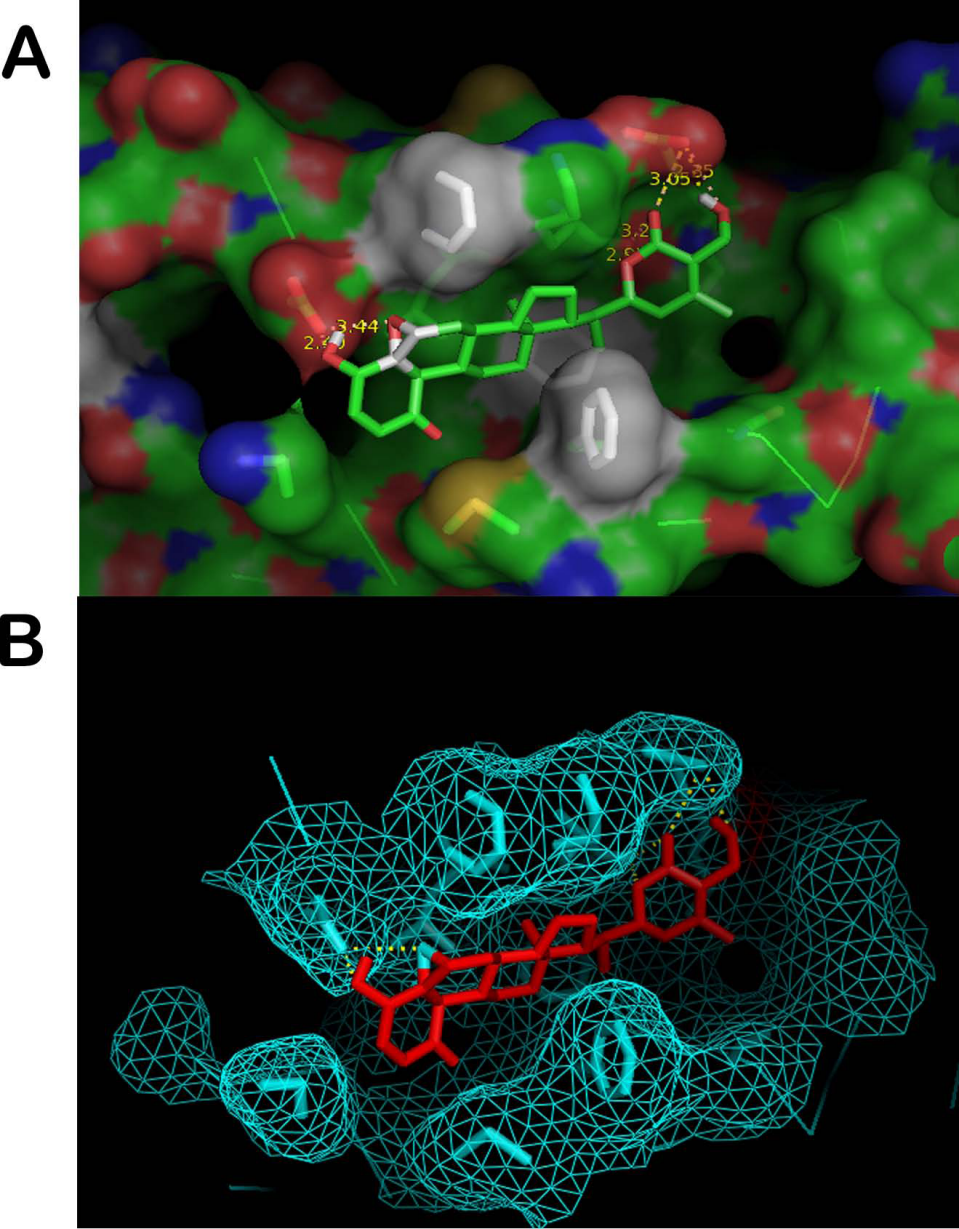
**Docking representations of Withaferin A.** (A) Ligand docked into the NEMO receptor cavity (B) Docked Ligand inside the pocket of NEMO receptor mesh.

One possible mode of action which is proposed here for WA to act as a NF-κB activation suppressor is by non-formation/disruption of the complex between NEMO and IKKβ. In order to explore the possibility of non-formation of the complex, we first carried out molecular docking studies with only the NEMO chains of the protein crystal structure. Before docking to be carried out, the structures of receptor macromolecules were minimized in Steepest Descent and Conjugate Gradient methods using Accelrys Discovery studio. Macromolecular receptors minimized using Conjugate Gradient have comparatively lower potential energy values than those obtained from Steepest Descent and are thus utilized further for carrying out the docking studies. Figure 3A shows the docked ligand WA to the selective NEMO receptor. WA gets buried inside the pocket of NEMO as depicted by mesh representation in Figure 3B. For this particular configuration the binding energy of WA with NEMO is -9.44 Kcal/mol (Table 1).

**Table 1:** Energies obtained after docking of withaferin A into NEMO

Property	Quantity
Binding Energy	-9.44 Kcal/mol
Intermolecular energy	-9.68 Kcal/mol
Total internal energy	-1.13 Kcal/mol

**Figure 4:**
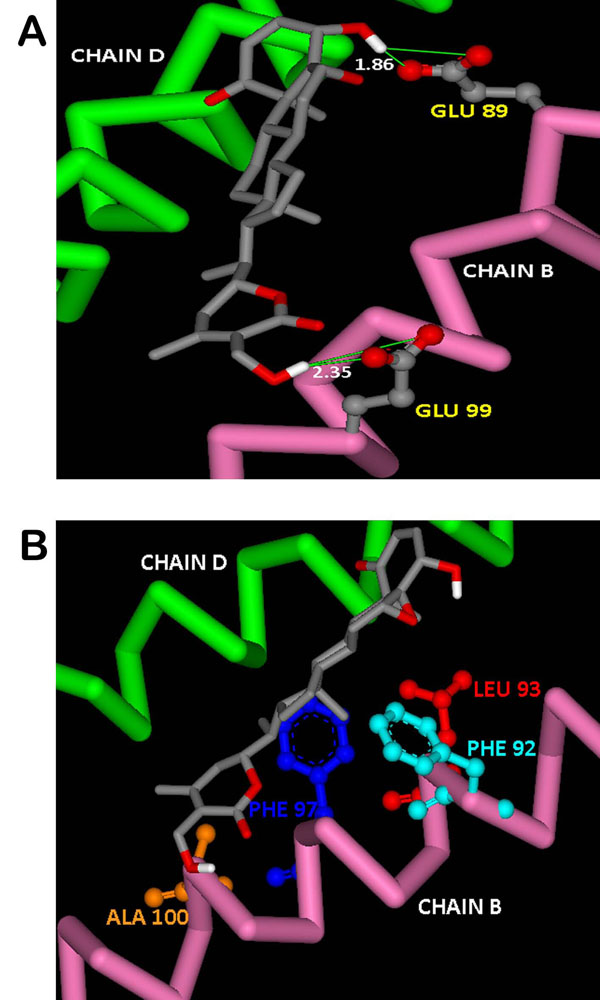
**Interactions of docked withaferin A with NEMO receptor.** (A) H-Bond interactions of the docked ligand with NEMO residues. (B) Docked withaferin A forming vdw interactions with the hydrophobic residues of NEMO.

The binding of WA to NEMO is characterized by H-bonding between a terminal hydroxyl group of the ligand and the side chain carboxyl group of Glu 89 (Figure 4A). The residue Glu 89 has been reported as one of the critical residues being involved in H-bond formation with Ser 733 of IKKβ resulting in complex formation with NEMO [25]. The lengths of the two H-bonds formed are 1.86 and 2.93 Å. The other end of WA also forms H-bond with the side chain carboxyl group of Glu 99 with a bond distance of 2.35 Å. It has also been reported that the residues Phe 92, Leu 93, Met 94, Phe 97, Ala 100 and Arg 101 of NEMO are responsible for intermolecular hydrophobic interactions. In the present docked structure, WA is forming van der Waals interactions with Phe 92, Leu 93, Phe 97 and Ala 100 of NEMO (Figure 4B). These non-covalent interactions help stabilize the binding of the ligand with the macromolecule by lowering the energy. Strong interactions formed by WA with these particular residues would result in building steric as well as thermodynamic barriers to the incoming IKKβ subunits, thus providing hindrance for binding to NEMO at these particular residues. This obstruction would pave way to unlikely complex formation capability of NEMO to IKKβ yielding in either the formation of a deformed complex or at extreme no complex formation at all. Moreover the deformed complex would be thermodynamically much less stable as compared to the native complex owing to the non-availability of WA occupied hydrophobic interaction forming residues of NEMO. In both cases the NEMO/IKKβ complex would not get assembled to its catalytically active form. The absence of active IKK multisubunit complex would prevent degradation of IκB proteins, as the IκB proteins would not get phosphorylated by IKK. This would ultimately lead to non-release of NF-κB and its further translocation to the nucleus thus arresting its nefarious acts.

### Flexible docking of WA into active NEMO/IKKβ complex

**Figure 5:**
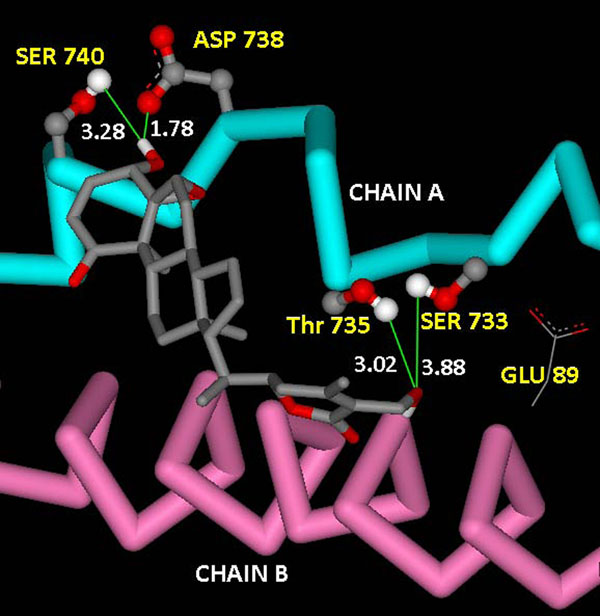
**H-bonding interactions present in the docked complex of withaferin A with active NEMO/IKKβ complex using flexible docking.** Polar hydroxyl terminal group of withaferin A captivates hydroxyl group of SER 733 to form H-bond with itself, thus disrupting the earlier present SER 733 - GLU 89 H-bond.

**Figure 6:**
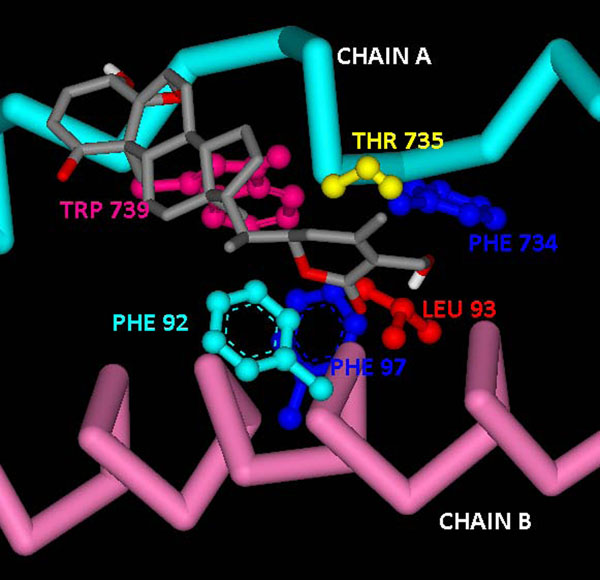
Hydrophobic interactions present in the complex obtained from docking of withaferin A into active NEMO/IKKβ complex using flexible docking

Docking of WA performed on the catalytically active NEMO/IKKβ complex also provided interesting results. The binding of WA to NEMO provides significant evidence in support of the proposed mechanism of NF-κB activation suppression by inhibition or disruption of active NEMO/IKKβ complex formation. Four clusters were obtained for Genetic Algorithm run for generation of twenty models. Large negative binding energies were obtained for all the clusters as evident from Table 2. The lowest binding energy was more than twice as lower as that of the energy obtained from binding of WA to the segregated NEMO receptor. As shown in Figure 5 for Cluster 1, the hydroxyl group of WA disrupts SER733:GLU89 H-bond present in the active complex by itself hydrogen bonding with SER733 hydroxyl group. The same hydroxyl group of the ligand is also involved in H-bonding with THR735, which is one of the critical residues in complex formation. The other terminal hydroxyl group of WA is also involved in forming H-bonds with SER740 and ASP738 of chain A. NEMO and IKKβ chains of active NEMO/IKKβ complex are held together by strong intermolecular hydrophobic interactions between LEU93:PHE734, THR735:PHE92, PHE734:MET94, TRP739:PHE97, TRP741:ALA100, TRP741:ARG101. But withefrin A disrupts majority of these hydrophobic interactions by placing itself in between the binding chains, As depicted in Figure 6 for Cluster 1, WA itself starts forming strong intermolecular interactions with these critical residues.

**Table 2:** Clustering results obtained from docking of withaferin A into NEMO/IKKβ complex

Receptor	No. of AutoDock clusters ^a, b^	Cluster rank^b^	No. of structures in the cluster	Lowest binding energy of cluster	Energy range within cluster
		1	8	-19.33	-19.33 to -17.24
NEMO/IKKβ complex	4 (20)	2	8	-19.12	-19.12 to -16.53
		3	3	-18.6	-18.6 to -16.99
		4	1	-18.33	-18.33

^a^ Number of GA runs are shown in parentheses

^b^ Clustering is done with RMS tolerance of 5.0 Å

**Figure 7:**
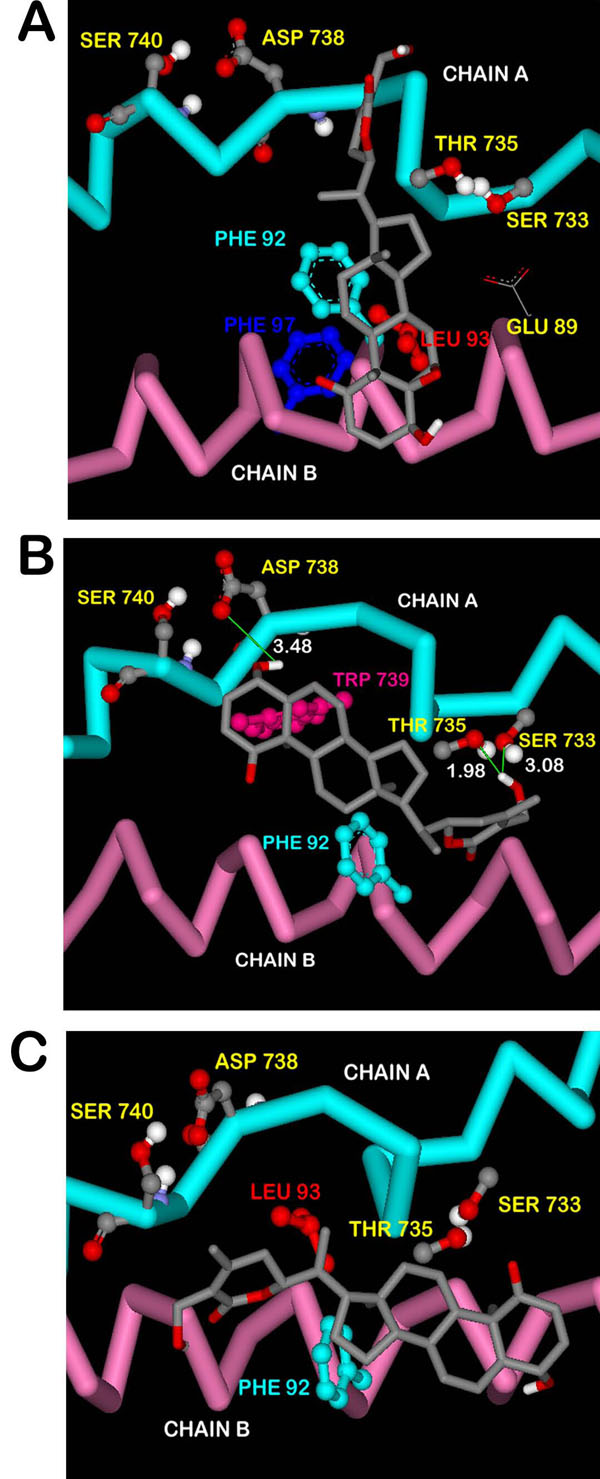
**Interactions of docked withaferin A with NEMO/IKKβ in the representative structures of different clusters using flexible docking.** (A) Cluster 2 – no hydrogen bond formation takes place (B) Cluster 3 – formation of few hydrogen bonds in addition to weak van der Waals interactions (C) Cluster 4 – no hydrogen bond formation in addition to weak van der Waals interactions.

The energy values for the different clusters shown in Table 2 can be explained on the basis of the representative cluster structures, which are the ones having lowest energy in the respective cluster (Figure 7). All the hydrogen bonds being formed in cluster 1 (Ser 733, Thr 735, Asp 738 and Ser 740) were lost in cluster 2 (Figure 7A) which explains the increase in energy for this cluster as compared to cluster 1. But the amount of energy lowered is not that much significant owing to the presence of strong van der Waals interactions with Phe 92, Leu 93 and Phe 97 of chain B. In cluster 3 (Figure 7B), though there is formation of few hydrogen bonds with residues of chain A, the van der Waals interactions are also markedly reduced resulting in further decrease in energy. In cluster 4 (Figure 7C), due to the absence of any hydrogen bond formation in addition to very weak van der Waals interactions, the energy is recorded as minimum of all the four clusters.

The results obtained (Figure 5 &6) from docking of WA into the active complex, with a majority of H-bond and hydrophobic interaction forming residues kept flexible, clearly show that WA blocks the intermolecular hydrophobic interactions between NEMO and IKKβ at the residues which are significantly involved in formation of the active complex. The large value of binding energy for Cluster 1(-19.33 Kcal/mol) involved in binding of WA to the complex consolidates the thermodynamic stability of the binding. These results substantiate the hypothesis that WA possess the potential to disarray the active complex by disrupting the stability of attachment of NEMO and IKKβ chains, being accounted by hydrophobic and H-bond interactions.

### MD simulations in water

**Figure 8:**
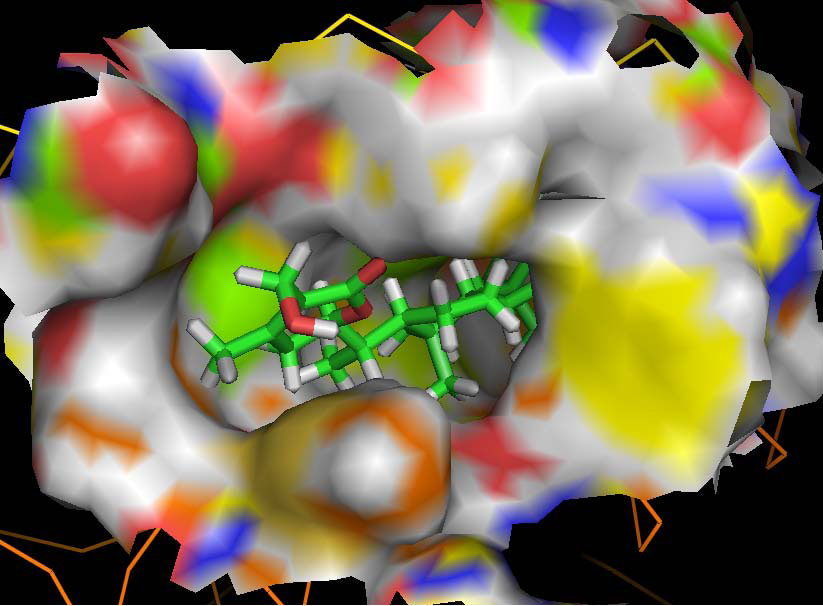
Docking representation of the drug WA inside the cavity of NEMO/IKKβ obtained using ParDOCK.

**Figure 9:**
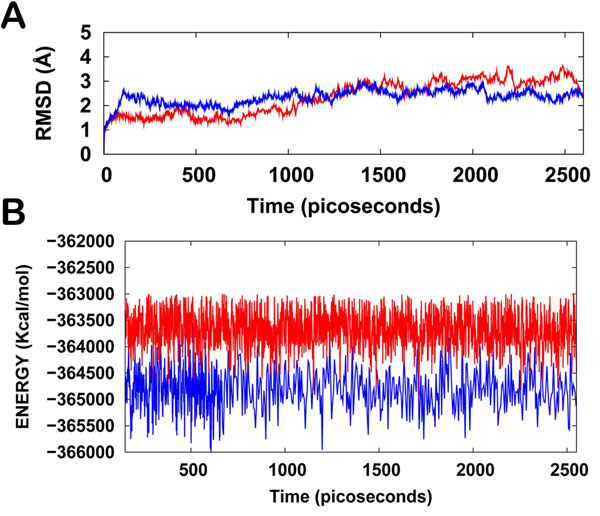
**(A) Plot of root mean square deviation (RMSD) of Cα of NEMO/IKKβ (protein) and NEMO/IKKβ/WA (complex).** RMSDs were calculated using the initial structures as templates. For protein (red) the reference is the PDB structure and for complex (blue) the reference is the initial model. The trajectories were captured every 1 ps until the simulation time reached 2600 ps. **(B) Plot of total energy of NEMO/IKKβ and NEMO/IKKβ/WA (complex)** The energy trajectories of both the protein (red) and the complex (blue) are stable over the entire length of simulation time.

**Figure 10:**
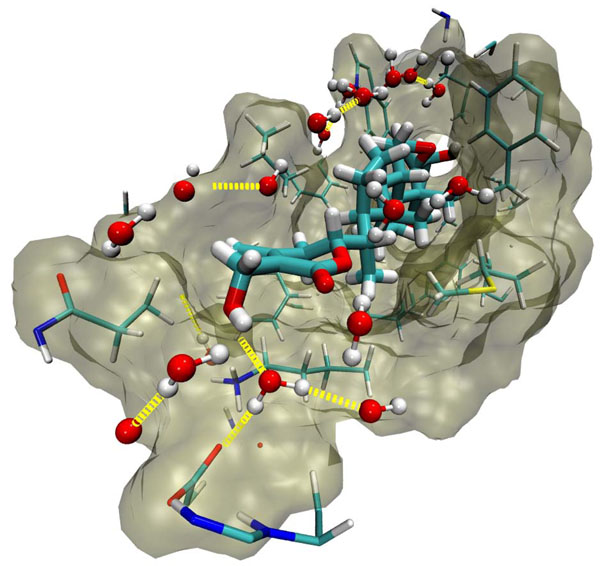
**A simulated snapshot of WA/NEMO/IKKβ complex showing water molecules coordinating the ligand and the protein.** The water molecules act as intermediate bridges facilitating the binding of WA to NEMO/IKKβ.

**Figure 11:**
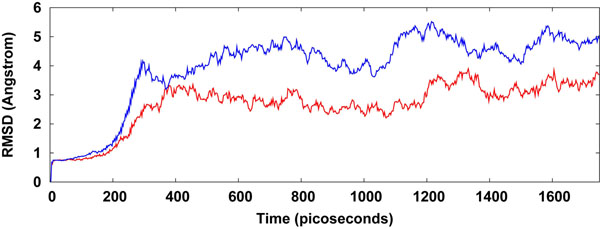
**Plot of root mean square deviation (RMSD) of Cα of NEMO (protein) and NEMO/WA (complex).** RMSDs were calculated using the initial structures as templates. For protein (red) the reference is the PDB structure and for complex (blue) the reference is the initial model. The trajectories were captured every 1 ps until the simulation time reached 1750 ps.

The NEMO/IKKβ/WA protein-drug binding complex with the binding energy of -9.33 kcal/mol obtained using ParDOCK (Figure 8) was used for carrying out MD simulations. After the MD simulation, we calculated RMSDs between Cα of NEMO/IKKβ complex trajectories recorded every 1 ps and Cα of their X-ray crystal structure. The RMSDs for the trajectories of the NEMO/IKKβ complexed with WA were also calculated using its initial model as a reference structure. The results in Figure 9A show that the RMSD of the complex has achieved a stationary phase during the later stage of the simulation and is always less than 3 Å for the entire simulation length suggesting the stability of the complex, while the RMSDs of the protein from its initial X-ray PDB structure kept increasing. It was also found that the energy of the complex (blue) is always lower than that of the protein (red) alone throughout the length of the simulation (Figure 9B). This rule out the possibility of the complex getting activated in the presence of WA. It was also observed that the water molecules act as intermediate bridges thus playing a vital role in facilitating the binding of ligand to the protein (Figure 10). The MD simulations were also carried out for WA complex with NEMO. It was observed that RMSD for this complex kept increasing with marked fluctuations (Figure 11). Thus the WA complex with NEMO alone seems to be quite unstable. The simulation length used in the entire study were long enough to allow rearrangement of side chains of the native as well as the drug complexed protein to find their most stable binding mode. In conclusion, the present MD simulations made clear the dynamic structural stability of NEMO/IKKβ in complex with the drug WA, together with the inhibitory mechanism. These results would be valuable for further designing non-covalent type inhibitors with high specificity and potent activity.

## Conclusions

NF-κB is one of the most attractive topics in current biological, biochemical, and pharmacological research, and in the recent years the number of studies focusing on its inhibition/regulation has increased manifolds. Small ligands (both natural and synthetic) are gaining particular attention in this context. Our computational analysis, provided a rationalization of the ability of naturally occurring WA to alter the NF-κB signalling pathway. The large value of binding energy involved in binding of WA to the active NEMO/IKK complex consolidates the thermodynamic stability of the binding. Our docking results obtained substantiate the hypothesis that WA has the potential to inhibit the formation of active NEMO/IKK complex by either resulting in non-formation of the complex or by disrupting the stability of attachment of NEMO to IKKβ chains. Conclusively our results strongly suggest that withaferin A is a potent anticancer agent as ascertained by its potent NF-κB modulating capability.

## Authors' contributions

AG, VSB and DS designed the methods and experimental setup. AG carried out the implementation of the various methods. AS and AP assisted AG in this process. AG and DS wrote the manuscript. All authors have read and approved the final manuscript.

## Competing interests

The authors declare that they have no competing interests.

## References

[B1] Hayden MS Ghosh S Signaling to NF-kappa B Gene Dev 2004 18 18 2195 2224 10.1101/gad.1228704 15371334

[B2] Baldwin AS Sharp PA Binding of a Nuclear Factor to a Regulatory Sequence in the Promoter of the Mouse H-2kb Class-I Major Histocompatibility Gene Mol Cell Biol 1987 7 1 305 313 3561391 10.1128/mcb.7.1.305-313.1987 PMC365070

[B3] Barnes PJ Nuclear factor kappa B Int J Biochem Cell B 1997 29 6 867 870 10.1016/S1357-2725(96)00159-8 9304801

[B4] Hiscott J Kwon H Genin P Hostile takeovers: viral appropriation of the NF-kappa B pathway J Clin Invest 2001 107 2 143 151 10.1172/JCI11918 11160127 PMC199181

[B5] Amit S Ben-Neriah Y NF-kappa B activation in cancer: a challenge for ubiquitination- and pro-teasome-based therapeutic approach Semin Cancer Biol 2003 13 1 15 28 10.1016/S1044-579X(02)00096-2 12507553

[B6] Karin M Delhase M The I kappa B kinase (IKK) and NF-kappa B: key elements of proinflamma-tory signalling Semin Immunol 2000 12 1 85 98 10.1006/smim.2000.0210 10723801

[B7] Ghosh S Karin M Missing pieces in the NF-kappa B puzzle Cell 2002 109 S81 S96 10.1016/S0092-8674(02)00703-1 11983155

[B8] Winters M Ancient medicine, modern use: Withania somnifera and its potential role in integrative oncology Alternative Medicine Review 2006 11 4 269 277 17176166

[B9] Matsuda H Murakami T Kishi A Yoshikawa M Structures of withanosides I, II, III, IV, V, VI, and VII, new withanolide gly-cosides, from the roots of Indian Withania somnifera Dunal. and inhibitory activity for tachyphylaxis to clonidine in isolated guinea-pig ileum Bioorgan Med Chem 2001 9 6 1499 1507 10.1016/S0968-0896(01)00024-4 11408168

[B10] Ray A Gupta M Hertz W, Kerby G, Moore R, Steglich W, Tamm C Withasteroids, a growing group of naturally occurring steroidal lactones Progress in the chemistry of natural organic products 1994 63 New York: Springer-Verlag 1 106 10.1007/978-3-7091-9281-8_1 7851821

[B11] Alhindawi MK Alkhafaji SH Abdulnabi MH Antigranuloma Activity of Iraqi Withania-Somnifera J Ethnopharmacol 1992 37 2 113 116 10.1016/0378-8741(92)90069-4 1434685

[B12] Mishra L Singh B Dagenias S Scientific basis for the therapeutic use of Withania somnifera (ashwa-gandha): a review Altern Med Rev 2000 5 334 336 10956379

[B13] Owais M Sharad KS Shehbaz A Saleemuddin M Antibacterial efficacy of Withania somnifera (ashwagandha) an indige-nous medicinal plant against experimental murine salmonellosis Phytomedicine 2005 12 3 229 235 10.1016/j.phymed.2003.07.012 15830846

[B14] Bhattacharya A Ghosal S Bhattacharya SK Anti-oxidant effect of Withania somnifera glycowithanolides in chronic footshock stress-induced perturbations of oxidative free radical scavenging enzymes and lipid peroxidation in rat frontal cortex and striatum J Ethnopharmacol 2001 74 1 1 6 10.1016/S0378-8741(00)00309-3 11137343

[B15] Kulkarni SK George B Mathur R Protective effect of Withania somnifera root extract on electrographic ac-tivity in a lithium-pilocarpine model of status epilepticus Phytotherapy Research 1998 12 6 451 453 10.1002/(SICI)1099-1573(199809)12:6<451::AID-PTR328>3.0.CO;2-C

[B16] Furmanowa M Gajdzis-Kuls D Ruszkowska J Czarnocki Z Obidoska G Sadowska A Rani R Upadhyay SN In vitro propagation of Withania somnifera and isolation of withanolides with immunosuppressive activity Planta Med 2001 67 2 146 149 10.1055/s-2001-11494 11301861

[B17] Sharada AC Solomon FE Devi PU Udupa N Srinivasan KK Antitumor and radiosensitizing effects of withaferin a on mouse Ehrlich ascites carcinoma in vivo Acta Oncol 1996 35 1 95 100 10.3109/02841869609098486 8619948

[B18] Begum VH Sadique J Long-Term Effect of Herbal Drug Withania-Somnifera on Adjuvant In-duced Arthritis in Rats Indian J Exp Biol 1988 26 11 877 882 3248848

[B19] Oh JH Kwon TK Withaferin A inhibits tumor necrosis factor-alpha-induced expression of cell adhesion molecules by inactivation of Akt and NF-kappa B in human pulmonary epithelial cells Int Immunopharmacol 2009 9 5 614 619 10.1016/j.intimp.2009.02.002 19236958

[B20] Ichikawa H Takada Y Shishodia S Jayaprakasam B Nair MG Aggarwal BB Withanolides potentiate apoptosis, inhibit invasion, and abolish osteo-clastogenesis through suppression of nuclear factor-kappa B (NF-kappa B) ac-tivation and NF-kappa B-regulated gene expression Mol Cancer Ther 2006 5 6 1434 1445 10.1158/1535-7163.MCT-06-0096 16818501

[B21] Kaileh M Vanden Berghe W Heyerick A Horion J Piette J Libert C De Keukeleire D Essawi T Haegeman G Withaferin A strongly elicits I kappa B kinase beta hyperphosphorylation concomitant with potent inhibition of its kinase activity J Biol Chem 2007 282 7 4253 4264 10.1074/jbc.M606728200 17150968

[B22] Mathur S Kaur P Sharma M Katyal A Singh B Tiwari M Chandra R The treatment of skin carcinoma, induced by UVB radiation, using 1-oxo-5 beta,6 beta-epoxy-witha-2-enolide, isolated from the roots of Withania somnifera, in a rat model Phytomedicine 2004 11 5 452 460 10.1016/j.phymed.2003.05.004 15330502

[B23] Kuboyama T Tohda C Komatsu K Withanoside IV and its active metabolite, sominone, attenuate A beta(25-35)-induced neurodegeneration Eur J Neurosci 2006 23 6 1417 1426 10.1111/j.1460-9568.2006.04664.x 16553605

[B24] Tohda C Kuboyama T Komatsu K Search for natural products related to regeneration of the neuronal network Neurosignals 2005 14 1-2 34 45 10.1159/000085384 15956813

[B25] Rushe M Silvian L Bixler S Chen LL Cheung A Bowes S Cuervo H Berkowitz S Zheng T Guckian K Structure of a NEMO/IKK-associating domain reveals architecture of the interaction site Structure 2008 16 5 798 808 10.1016/j.str.2008.02.012 18462684

[B26] Berman HM Westbrook J Feng Z Gilliland G Bhat TN Weissig H Shindyalov IN Bourne PE The Protein Data Bank Nucleic Acids Res 2000 28 1 235 242 10.1093/nar/28.1.235 10592235 PMC102472

[B27] NCBI-PubChem Compound database http://pubchem.ncbi.nlm.nih.gov/

[B28] Morris GM Goodsell DS Halliday RS Huey R Hart WE Belew RK Olson AJ Automated docking using a Lamarckian genetic algorithm and an empiri-cal binding free energy function J Comput Chem 1998 19 14 1639 1662 10.1002/(SICI)1096-987X(19981115)19:14<1639::AID-JCC10>3.0.CO;2-B

[B29] Dym O Xenarios I Ke HM Colicelli J Molecular docking of competitive phosphodiesterase inhibitors Mol Pharmacol 2002 61 1 20 25 10.1124/mol.61.1.20 11752202

[B30] Rao MS Olson AJ Modelling of Factor Xa-inhibitor complexes: A computational flexible docking approach Proteins 1999 34 2 173 183 10.1002/(SICI)1097-0134(19990201)34:2<173::AID-PROT3>3.0.CO;2-F 10022353

[B31] Goodsell DS Morris GM Olson AJ Automated docking of flexible ligands: Applications of AutoDock J Mol Recognit 1996 9 1 1 5 10.1002/(SICI)1099-1352(199601)9:1<1::AID-JMR241>3.0.CO;2-6 8723313

[B32] Hetenyi C van der Spoel D Efficient docking of peptides to proteins without prior knowledge of the binding site Protein Sci 2002 11 7 1729 1737 10.1110/ps.0202302 12070326 PMC2373668

[B33] Cornell WD Cieplak P Bayly CI Gould IR Merz KM Ferguson DM Spellmeyer DC Fox T Caldwell JW Kollman PA A second generation force field for the simulation of proteins, nucleic acids, and organic molecules J Am Chem Soc 1996 118 9 2309 2309 10.1021/ja955032e

[B34] Morris GM Goodsell DS Huey R Olson AJ Distributed automated docking of flexible ligands to proteins: Parallel applications of AutoDock 2.4 J Comput Aid Mol Des 1996 10 4 293 304 10.1007/BF00124499 8877701

[B35] DeLano W The PyMOL Molecular Graphics System 2002 In San Carlos CA: DeLano Scientific 2002

[B36] Gupta A Gandhimathi A Sharma P Jayaram B ParDOCK: An all atom energy based Monte Carlo docking protocol for protein-ligand complexes Protein Peptide Lett 2007 14 7 632 646 10.2174/092986607781483831 17897088

[B37] Case DA Darden TA Cheatham TE Simmerling CL Wang J Duke RE Luo R Crowley M Walker RC Zhang W AMBER 10 University of California 2008

[B38] Jorgensen WL Chandrasekhar J Madura JD Impey RW Klein ML Comparison of Simple Potential Functions for Simulating Liquid Water J Chem Phys 1983 79 2 926 935 10.1063/1.445869

[B39] Jakalian A Bush BL Jack DB Bayly CI Fast, efficient generation of high-quality atomic Charges. AM1-BCC model: I. Method J Comput Chem 2000 21 2 132 146 10.1002/(SICI)1096-987X(20000130)21:2<132::AID-JCC5>3.0.CO;2-P 12395429

[B40] Berendsen HJC Postma JPM Vangunsteren WF Dinola A Haak JR Molecular-Dynamics with Coupling to an External Bath J Chem Phys 1984 81 8 3684 3690 10.1063/1.448118

[B41] Ryckaert JP Ciccotti G Berendsen HJC Numerical-Integration of Cartesian Equations of Motion of a System with Constraints - Molecular-Dynamics of N-Alkanes J Comput Phys 1977 23 3 327 341 10.1016/0021-9991(77)90098-5

[B42] Essmann U Perera L Berkowitz ML Darden T Lee H Pedersen LG A Smooth Particle Mesh Ewald Method J Chem Phys 1995 103 19 8577 8593 10.1063/1.470117

